# Association of estrogen receptor-α and progesterone receptor A expression with hormonal mammary carcinogenesis: role of the host microenvironment

**DOI:** 10.1186/bcr1660

**Published:** 2007-03-06

**Authors:** Guadalupe Montero Girard, Silvia I Vanzulli, Juan Pablo Cerliani, María Cecilia Bottino, Julieta Bolado, Jorge Vela, Damasia Becu-Villalobos, Fernando Benavides, Silvio Gutkind, Vyomesh Patel, Alfredo Molinolo, Claudia Lanari

**Affiliations:** 1 Instituto de Investigaciones Oncológicas, Academia Nacional de Medicina, 3092 Las Heras, Buenos Aires 1425, Argentina; 2 Instituto de Biología y Medicina Experimental, Consejo Nacional de Investigaciones Científicas y Técnicas, 2490 V de Obligado, Buenos Aires 1428, Argentina; 3 Department of Carcinogenesis, Science Park Research Division, The University of Texas MD Anderson Cancer Center, Park Road 1C, Science Park, Smithville, Texas 78957, USA; 4 Oral and Pharyngeal Cancer Branch, National Institute of Dental and Craniofacial Research, National Institutes of Health, Rockville Pike, Bethesda, Maryland 20892, USA

## Abstract

**Introduction:**

Medroxyprogesterone acetate (MPA) induces estrogen receptor (ER)-positive and progesterone receptor (PR)-positive ductal invasive mammary carcinomas in BALB/c mice. We sought to reproduce this MPA cancer model in C57BL/6 mice because of their widespread use in genetic engineering. Within this experimental setting, we studied the carcinogenic effects of MPA, the morphologic changes in mammary glands that are induced by MPA and progesterone, and the levels of ER and PR expression in MPA-treated and progesterone-treated mammary glands. Finally, we evaluated whether the differences found between BALB/c and C57BL/6 mouse strains were due to intrinsic differences in epithelial cells.

**Methods:**

The carcinogenic effect of MPA was evaluated in C57BL/6 mice using protocols proven to be carcinogenic in BALB/c mice. In addition, BALB/c and C57BL/6 females were treated with progesterone or MPA for 1 or 2 months, and mammary glands were excised for histologic studies and for immunohistochemical and Western blot evaluation of ER and PR. Hormone levels were determined by radioimmunoassay. Isolated mammary epithelial cells were transplanted into cleared fat pads of 21-day-old female Swiss *nu*/*nu *mice or control congenic animals.

**Results:**

MPA failed to induce mammary carcinomas or significant morphologic changes in the mammary glands of C57BL/6 mice. The expression of ER-α and PR isoform A in virgin mice was surprisingly much higher in BALB/c than in C57BL/6 mammary glands, and both receptors were downregulated in progestin-treated BALB/c mice (*P *< 0.05). PR isoform B levels were low in virgin control mice and increased after progestin treatment in both strains. ER-β expression followed a similar trend. No differences in hormone levels were found between strains. Surprisingly, the transplantation of the epithelial mammary gland cells of both strains into the cleared fat pads of Swiss (*nu*/*nu*) mice abolished the mammary gland morphologic differences and the ER and PR differences between strains.

**Conclusion:**

C57BL/6 mammary glands are resistant to MPA-induced carcinogenesis and to hormone action. MPA and progesterone have different effects on mammary glands. Low ER-α and PR-A levels in untreated mammary glands may be associated with a low-risk breast cancer profile. Although we cannot at this time rule out the participation of other, untested factors, our findings implicate the stroma as playing a crucial role in the strain-specific differential hormone receptor expression and hormone responsiveness.

## Introduction

Breast cancer is the most common malignancy diagnosed among women worldwide, and it is the second leading cause of cancer mortality [1]. One of the hallmarks of the disease is the expression of estrogen receptor (ER) and progesterone receptor (PR), and it is this expression that ultimately drives prognosis and treatment approaches.

During the past 50 years, the experimental study of breast cancer has been approached utilizing many different *in vivo *as well as *in vitro *models, some of which have been used widely [2,3]: the 9,10-dimethyl-1,2-benzanthracene [4,5] and *N*-methyl-*N*-nitrosourea induced mammary tumors in rats [6]; mouse mammary tumor virus (MMTV)-induced carcinomas [7]; human breast cancer cell lines growing in cell culture [8-11] or as xenografts [12]; transgenic and knockout animals [13,14]; and, more recently, conditional knockout and inducible transgenic mice [15]. Genetically engineered mice have been used extensively to model human breast cancer as well as to dissect the molecular pathways of tumorigenesis [16], and they have contributed much to our understanding of how cancer evolves. Interestingly, most of the genetically modified mice available for the study of breast cancer do not express ER and PR [2], leaving few models available in which to address hormone-dependent tumor progression specifically.

Medroxyprogesterone acetate (MPA) induces ductal mammary carcinomas in BALB/c mice that express ER and PR [17,18]. During preliminary studies, conducted to investigate strains that may be susceptible to MPA other than BALB/c, we found C57BL/6 to be resistant to the standard MPA dosage that is used to develop mammary carcinomas. This strain had already been reported to be resistant to mammary tumorigenesis induced by MMTV [7], urethane [19] and γ radiation [20,21]. Interestingly, however, most of the genetically modified mammary tumor models have been developed in a C57BL/6 or FVB background [16], without any previous experimental evidence to indicate that these strains were suitable for the study of mammary tumorigenesis, in terms of susceptibility to the more standard hormonal/chemical approaches.

With this in mind, we decided to study further the resistance of the C57BL/6 strain to hormonal carcinogenesis, and to advance the characterization of possible underlying differences in hormone responsiveness. In this study we demonstrated that C57BL/6 mammary glands are resistant not only to MPA carcinogenesis but also to hormone-induced mammary gland development. The higher responsiveness of BALB/c mice was associated with higher levels of expression of ER-α and PR isoform A, suggesting that a high ER-α and PR-A profile may be related to breast cancer susceptibility. Finally, we were able to demonstrate that this specific strain difference disappeared when epithelial cells of either strain were transplanted into a different strain background, indicating that host factors, possibly stroma, may be responsible for the differential hormone responsiveness.

## Materials and methods

### Animals

Two month-old virgin female BALB/c mice (Instituto de Biología y Medicina Experimental, Buenos Aires, Argentina) and C57BL/6J mice (Instituto de Biología y Medicina Experimental and Academia Nacional de Medicina, Buenos Aires, Argentina) were used for these experiments, and Swiss-*Foxn1*^*nu*^*/Foxn1*^*nu *^(*nu*/*nu*) were purchased from La Plata Animal Facilities (Buenos Aires Province, Argentina). The animals were housed in groups of four per cage in an air-conditioned room at 20 ± 2°C under a 12-hour light/dark cycle, and given free access to food and tap water. Animal care and manipulation were in agreement with institutional guidelines and with the Guide for the Care and Use of Laboratory Animals [22]. Athymic nude mice were maintained under the same conditions in a germ-free environment.

### Tumor incidence: long-term experiments

#### Experiment 1

This experiment, conducted in C57BL/6 mice, was performed simultaneously with previously reported experiments in BALB/c mice, applying the same protocol originally used to induce mammary carcinomas [23,24]. A total of 38, 2-month-old mice, were subcutaneously implanted with 40 mg MPA depot (Medrosterona, Gador Laboratory, Buenos Aires, Argentina) every 2 months (four times) for 1 year; 31 control animals received only vehicle. The animals were evaluated twice weekly to detect the appearance of mammary tumors. This experiment was terminated after 1 year because of the emergence of severe skin ulcerations in MPA-treated C57BL/6 mice and in some of the control animals. It has been reported that aged C57BL/6 mice frequently develop spontaneous ulcerative dermatitis [25].

#### Experiment 2

To minimize the possibility of development of skin lesions, the experiment was repeated with a lower MPA dosage, which also proved to be carcinogenic in BALB/c mice [23]. Silastic pellets containing 40 mg MPA (Sigma Co, St Louis, MO, ISA) were implanted subcutaneously in 39 virgin, 2-month-old, C57BL/6 female mice, and replaced 6 months later; an equal number of control animals were implanted with control blank silastic pellets. The mice were evaluated daily, and those that developed skin lesions were isolated to prevent further damage, but continued to be evaluated as part of the experiment. The animals were weighed every week and followed for 14 months. Vaginal smears were obtained from selected mice in control and MPA-treated groups to confirm hormone action. After 6 months, MPA-treated mice started to cycle again and MPA pellets were replaced. Every 2 months two mice from both groups were killed for histologic evaluation of mammary gland hormone responsiveness. At the end of the experiments both control and treated animals were killed and mammary glands fixed in formalin (right 4th gland), prepared for whole mounts (left 4th gland), or frozen for Western blot studies.

### Short-term experiments

#### Mammary gland and salivary gland morphologic studies

A total of 24 BALB/c and 24 C57BL/6 virgin females (2-month-old) were implanted subcutaneously with 20 mg MPA, progesterone (Sigma Co.), or control blank silastic pellets (four mice per group). After 4 or 8 weeks the animals were killed, and salivary glands and the 4th right mammary glands were fixed in formalin and embedded in paraffin. Left mammary glands were processed for whole mounts. This experiment was repeated twice. Control animals were killed at diestrus. The morphologic features and the number of ducts, lateral branches, and lobules of the mammary glands of virgin untreated and progestin-treated mice were evaluated in hematoxylin and eosin stained slides and quantified in 40× fields.

#### Whole mount studies

The mammary glands were excised together with the attached skin, stretched and pinned down to a cork slab, and fixed in 10% buffered formalin for 24 hours. After fixation, the glands were dissected and extensively washed with distilled water, and lipids were removed by immersing in acetone for 2 days, at room temperature. After hydration with decreasing concentrations of ethanol, the tissues were left overnight in water and stained with toluidine blue (0.025% in water) for 40 min followed by thorough washings with distilled water. The staining was adjusted by destaining, if necessary, with methanol for 30 min and 70% ethanol for other 30 min, under microscopic observation. After washing with distilled water the tissues were fixed in ammonium molybdate for 30 min, washed again, dehydrated in ethanol with increasing concentrations, and transferred to xylene overnight before mounting.

#### Immunohistochemistry

Paraffin-embedded sections were reacted with various antibodies using the avidin biotin peroxidase complex technique (Vectastain Elite ABC kit; Vector Laboratories, Burlingame, CA, USA). Briefly, after dewaxing and hydrating, endogenous peroxidase activity was inhibited using 3% H_2_O_2 _in distilled water. Blocking solution (2% normal goat serum) was used before specific antibody. Polyclonal antibodies to ER-α (MC-20; Santa Cruz Biotechnology, Santa Cruz, CA, USA), ER-β (chicken antibody kindly provided by Jan-Åke Gustafsson, Karolinska Institutet, Novum, Sweden), and PR A (C-20; Santa Cruz Biotechnology) and the monoclonal antibody to PR-B (Ab 6; Neomarkers, Union City, CA) were used at 1:100 dilution and incubated overnight at 4°C. After incubation with the appropriate secondary antibodies (Vector Laboratories) the reactions were developed with 3-3'diaminobenzidine 0.30% in phosphate-buffered saline with H_2_O_2 _to a final concentration of 0.5%, under microscopic control. Specimens were counterstained with hematoxylin, dehydrated, and mounted. To quantify ER and PR, 1500 epithelial cells per slide were counted.

#### Western blots

Mammary glands were homogenized with a polytron at setting 50 with three bursts of 5 s at a 1:4 ratio of tissue to TEDGS (50 mmol/l Tris [pH 7.4], 7.5 mmol/l EDTA, 0.5 mmol/l dithiothreitol, 10% glycerol, and 0.25 mol/l sucrose) buffer. Protease inhibitors [0.5 mmol/l phenylmethylsulfonylfluoride (PMSF), 0.025 mmol/l CBZ-L-phenylalanine choromethylketone (ZPCK), 0.0025 mmol/l N-alpha-p-tosyl-L-lysine choro-methylketone (TLCK), 0.025 mmol/l N-alpha-p-tosyl-L-phenylalaninechlomethylketone (TPCK), 0.025 mmol/l N-alpha-p-tosyl-L-argininemethylester (TAME)] were added before preparing the extracts. The homogenate was centrifuged for 10 min at 3,300 rpm at 4°C, and the nuclei on the pellet were washed in TEDGS buffer plus 0.01% NP-40. The nuclei were resuspended in TEDGS containing 0.4 mol/l KCl and incubated at 4°C for 30 min, and the nuclear homogenate was centrifuged at 12,000 rpm for 20 min at 4°C. The nuclear extract was diluted 1:2 in TEDGS buffer with 30% glycerol, to reduce the salt concentration. Proteins were quantified using the method proposed by Lowry and coworkers [26].

The samples (100 μg total protein/lane) were separated on 8% (PR) or 10% (ER) discontinuous polyacrylamide gels (SDS-PAGE) using Laemmli's buffer system [27]. The proteins were dissolved in sample buffer (6 mmol/l Tris [pH 6.8], 2% SDS, 0.002% bromophenol blue, 20% glycerol, and 5% mercaptoethanol) and boiled for 4 min. After electrophoresis, they were blotted onto a nitrocellulose membrane and blocked overnight in 5% dry skimmed milk dissolved in phosphate-buffered saline-Tween (PBST) 0.1% (0.8% NaCl, 0.02% KCl, 0.144% Na_2_PO_4_, 0.024% KH_2_PO_4 _[pH 7.4], 0.1% Tween 20). The membranes were washed several times with PBST and then incubated with PR Ab-7/hPRa 7 (Ab7; Neomarkers, Union City, CA, USA) or C-19 (Santa Cruz), ER-α (MC-20; Santa Cruz), or actin (clone ACTN05; Neomarkers) at room temperature for 2 hours, at a concentration of 2 μg/ml in PBST. Blots were probed with sheep anti-mouse or donkey anti-rabbit immunoglobulin, horseradish peroxidase-conjugated whole antibody (Amersham Life Science, Buckinghamshire, UK). The luminescent signal was generated with the ECL Western blotting detection reagent kit (Amersham Pharmacia Biotech, Buckinghamshire, UK), and the blots were exposed to medical X-ray film (Curix RP1; Agfa Argentina, Florencio Varela, Buenos Aires) for 10 s to 5 min. Band intensity was not quantified if the film was saturated.

#### Effect of 17β-estradiol on mammary gland development

Four BALB/c and four C57BL/6 mice were implanted with 20 μg 17β-estradiol (E_2_) pellets, or E_2 _plus 20 mg MPA, or progesterone, and killed after 2 months. Mammary glands were processed as described above for morphologic studies.

#### Prolactin, growth hormone, and insulin-like growth factor I

BALB/c (*n *= 28) and C57BL/6 (*n *= 27), 2-month-old female mice were implanted subcutaneously with control blank silastic, MPA (40 mg), or progesterone (40 mg) pellets, and after 24 hours the animals were bled by retrorbital puncture under anesthesia. Control mice were bled at diestrus, as evaluated by vaginal smears. Prolactin and growth hormone were measured by radioimmunoassay using a kit provided by the National Institute of Diabetes and Digestive and Kidney Diseases (NIDDK; Dr AF Parlow, National Hormone and Pituitary Program, Torrance, CA, USA). The assays were performed using 10 μl serum samples in duplicate, and the results were expressed in terms of the reference preparation of mouse prolactin NIDDK-RP3 and mouse growth hormone AFP-107836 standards, respectively. Intra-assay and interassay coefficients of variation were 7.2% and 12.8%, and 8.4% and 13.2%, respectively [28]. For insulin-like growth factor (IGF)-I radioimmunoassay, serum samples (15 μl) and IGF-I standards were subjected to the acid-ethanol cryoprecipitation method, as previously described [28]. IGF-I was determined using an antibody (UB2-495) provided by Drs L Underwood and JJ Van Wyk, and distributed by the Hormone Distribution Program of the NIDDK. Recombinant human IGF-I (Chiron Corp., Emeryville, CA, USA) was used as radioligand and unlabeled ligand. The assay sensitivity was 6 pg per tube. Intra-assay and interassay coefficients of variation were 8.2% and 14.1%, respectively. Progesterone was determined by radioimmunoassay using an antibody kindly provided by Dr GD Niswender (Colorado State University, Fort Collins, CO, USA), and labeled hormone (progesterone [1,2,6,7 3H(N)]) was purchased from Dupont NEN, Boston, MA, USA. Assay sensitivity was 50 pg, and intra and interassay coefficients of variance were 7.5 and 11.9%, respectively.

#### Transplantation of BALB/c or C57BL/6 epithelial mammary cells into cleared fat pads from Swiss nude mice

Mammary glands from six C57BL/6 and six BALB/c 2-month-old female mice were excised and epithelial cells were isolated using the same protocol that we routinely use to isolate mammary tumor cells [29]. An equal number of cells (10^6 ^in 5 μl) was inoculated in the right (BALB/c) or left (C57BL/6) cleared fat pads of 21-day-old female *nu*/*nu *mice (Swiss background) that had previously been implanted subcutaneously with progesterone (20 mg) or MPA (20 mg) silastic pellet also containing 20 μg E_2_; four animals received blank silastic pellets, and four animals were used as a control ensuring that no epithelial cells remained after clearing the fat pads. The mammary glands were cleared according to standardized protocols [30,31]. Four BALB/c and four C57BL/6 mice were also used as control animals. After 30 days, mammary glands were excised, fixed in 4% buffered formaldehyde, and prepared for histologic evaluation. The experiment was repeated twice. To capture the entire area of interest, several partially overlapping high-definition images were taken from each case with a total magnification of 160×, using a real-time Nikon 1300 digital camera with the QImaging Pro software (MVIA, Inc., Monaca, PA, USA). The images were then aligned together using the PanaVue Image Assembler Software (PanaVue Co., Quebec City, Quebec, Canada). The resulting image was a high-quality panoramic picture of the lesion that allowed us to select the epithelial areas. Images were quantified using the Northern Eclipse Image Analysis Software (Empix Imaging, North Tonawanda, NY, USA) and expressed as the total epithelial area and the number of structures selected in each histologically defined area. To determine ER and PR expression in reconstituted mammary glands, the same procedures were performed in four untreated Swiss *nu*/*nu *mice, BALB/c or C57BL/6 mice.

#### Laser capture microdissection studies

Four to six 6 μm consecutive sections from normal mammary gland were obtained from paraffin-embedded tissue, dewaxed as described above, and stained with hematoxylin and eosin. Approximately 5 × 10^3 ^ductal epithelial cells were then microdissected using a PixCell II LCM system (Arcturus Engineering, Mountain View, CA, USA). Briefly, the cap was inserted into an Eppendorf tube containing digestion buffer (50 μl buffer containing 0.04% Proteinase K*, 10 mmol/l Tris-HCL [pH 8.0], 1 mmol/l EDTA, and 1% Tween-20). The tube was placed in an oven at 37°C to equilibrate and then placed upside down so that the digestion buffer contacts the tissue on the cap. The incubation continued overnight at 37°C and was then centrifuged for 5 min and the cap removed. The reaction was heated to 95°C for 8 min to inactivate the proteinase K. Samples were purified by means of the DNeasy Tissue System kit (Qiagen, Chatsworth, CA, USA) before PCR. We performed tests of the DNA samples by PCR amplifications with four informative microsatellite markers.

#### Microsatellite genotyping

Epithelial DNA was typed by PCR using a panel of four microsatellites (also known as simple sequence length polymorphisms) polymorphic between C57BL/6 and the outbred Swiss recipient nude females. The simple sequence length polymorphism primers (Mouse MapPairs) were purchased from Invitrogen (Carlsbad, CA, USA). An aliquot of 100 ng genomic DNA (5 μl of 20 ng/μl) was amplified in a 25 μl PCR reaction containing 2.5 μl 10× PCR buffer (15 mmol/l MgCl_2_), 0.5 μl 10 mmol/l dNTP, 1 μl of each primer (final concentration 180 μmol/l), 0.1 μl 5 U/μl Taq polymerase, and 14.9 μl sterile water. Hot start PCR (in which crucial reaction components are withheld until reaction temperatures are reached) was performed by means of FastStart Taq polymerase (Roche, Indianapolis, IN, USA). Initial denaturation (94°C for 6 min), 35 cycles (45 s at 94°C, 45 s at 55°C, and 30 s at 72°C), and final incubation at 72°C for 7 min were carried out using a Perkin-Elmer Model 9600 DNA thermal cycler (Applied Biosystems, Foster City, CA, USA). PCR products were separated by electrophoresis in 4% agarose gels, and stained with 0.5 μg/ml ethidium bromide.

### Statistical analysis

Differences between groups in the number of structures and stained nuclei were evaluated using analysis of variance (ANOVA) followed by the Tukey *t*-test to compare differences between experimental and control groups. To compare differences in body weight or prolactin, growth hormone, IGF-I, or progesterone serum levels between progestin-treated and untreated mice, a two-way ANOVA was used. The actuarial incidence of mammary tumor was calculated according to Kaplan-Meier distributions and log rank tests as described previously [23], and differences in tumor incidences were evaluated using χ^2 ^tests.

## Results

### Long term *in vivo *experiments

#### Tumor incidence

From a total of 47 BALB/c mice initially treated with MPA, 34 developed ductal infiltrating carcinomas, from which 13 arose during the first 12 months (27.6%). The mean latency was 52 weeks, and no mammary tumors were observed in untreated control animals [24]. MPA-treated C57BL/6 female mice did not develop mammary tumors, and neither did the control untreated animals (treated BALB/c versus treated C57BL/6 for 1 year; *P *< 0.001; data not shown). C57BL/6 mice treated with MPA developed severe skin ulcerations, and this arm of the experiment had to be stopped after a year.

**Figure 1:**
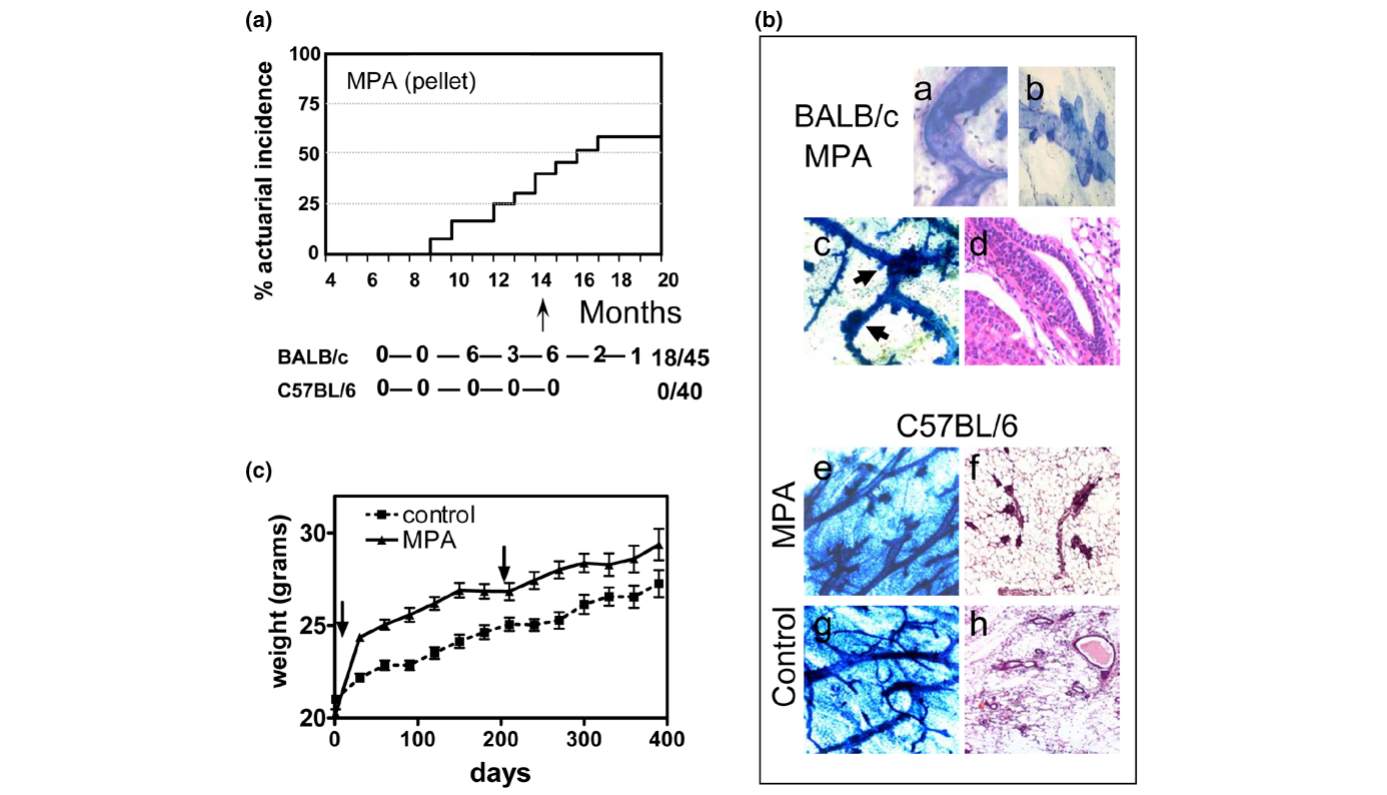
Tumor incidence, mammary gland alterations and body weight curves in MPA-treated mice. (a) Mammary tumor incidence. C57BL/6 mice were implanted with 40 mg MPA pellets that were replaced 6 months later; historical values of BALB/c using the same protocol were included for comparison. No mammary tumors appeared in MPA-treated or control C57BL/6 mice; control mice were implanted subcutaneously with silastic pellets. **(b) **Mammary gland alterations in MPA-treated BALB/c and C57BL/6 mice after one year of MPA treatment. The subpanels show the following: (a) a distorted and rotated ductal structure that appears to be forcibly stretched in areas (WM); (b) hyperplasia in the form of irregular paraductal proliferation (WM); (c) atypical ductal side branching showing areas of neoplastic growth (arrows; WM); (d) hematoxylin and eosin showing ductal hyperplasia; (e,f) WM and hematoxylin and eosin pictures showing nearly normal mammary glands of MPA-treated C57BL/6 mice; and (g,h) WM and hematoxylin and eosin pictures of C57BL/6 control mice. **(c) **Body weight curves of C57BL/6 mice implanted with 40 mg MPA (first arrow) or control blank silastic pellets that were replaced after 6 months (second arrow). All animals were weighed weekly and the results expressed as mean ± standard error. MPA-treated animals showed an increase in body weight that was already evident after the second week of treatment (*P *< 0.001; two-way ANOVA). ANOVA analysis of variance; MPA, medroxyprogesterone acetate; WM, whole mount.

In a second experiment, only C57BL/6 mice were treated using a protocol that had also proven to be carcinogenic in BALB/c animals, consisting of implanting 40 mg MPA pellets at the beginning of the experiment and replacing them after 6 months [23]. None of the animals in this group developed any mammary lesions, and after 14 months the experiment was terminated (Figure 1a).

#### Mammary gland morphology

MPA induces proliferative ductal lesions in BALB/c mice that are characterized by preneoplastic intraductal solid, papillary, or cribiform growth associated with an intense stromal reaction, with periductal fibrosis, an increase in mammary connective tissue, and moderate to intense mononuclear infiltration [32]. In whole mount studies, mammary glands from MPA-treated animals exhibited a gross distortion of the mammary gland architecture and developed hyperplasia in the form of paraductal branching (Figure 1b, top). Ductal hyperplasia was also a common finding. None of these lesions were observed in C57BL/6 MPA-treated mice (Figure 1b, bottom).

#### Effect of MPA on body weight

We previously observed a significant anabolic effect of MPA on the body weight of BALB/c mice [33,34] and, as shown in this study, a similar effect was observed in C57BL/6 mice (Figure 1c; two-way ANOVA, *P *< 0.001).

### Short term experiments

#### Mammary gland morphology

**Figure 2:**
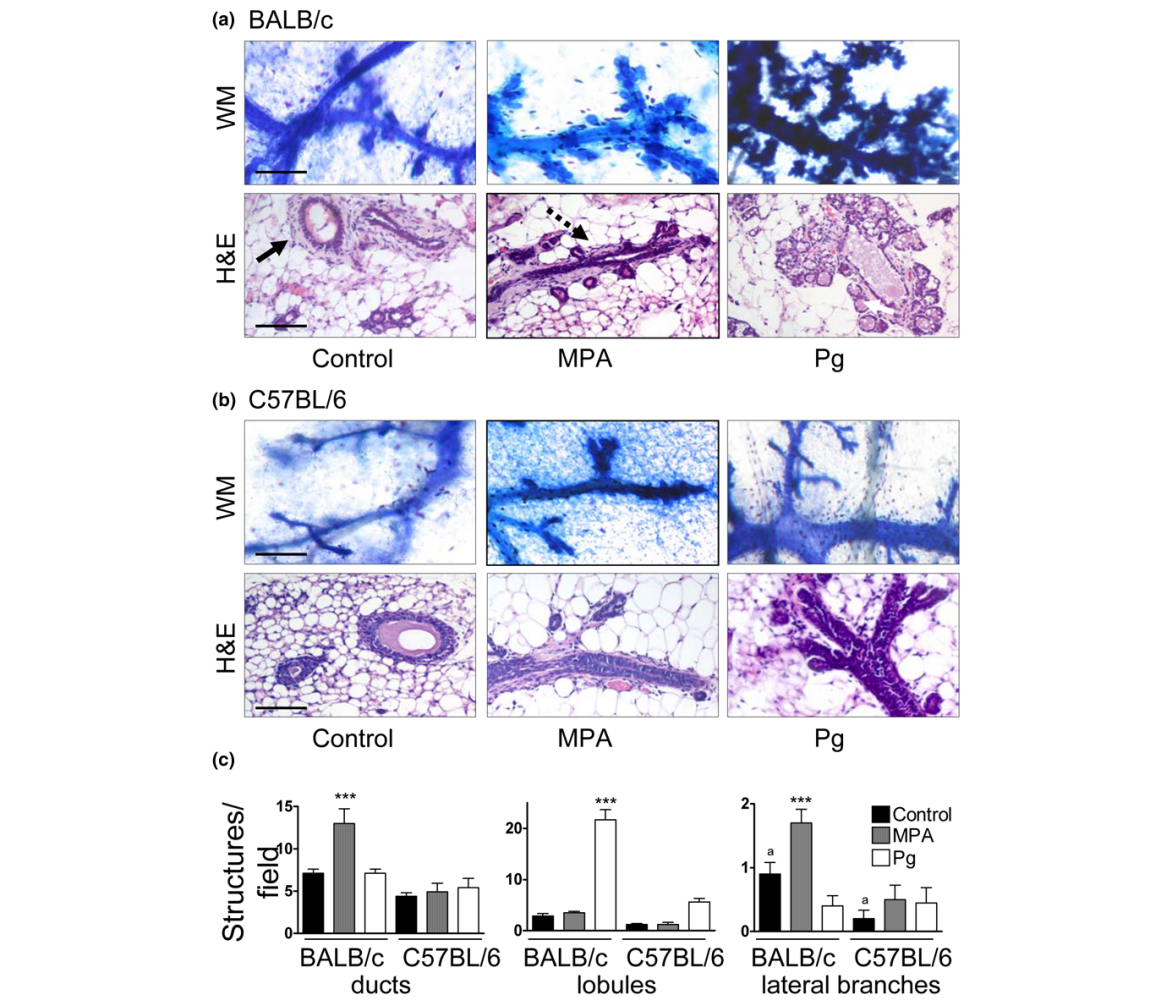
Mammary glands from progestin-treated mice. MPA (40 mg), Pg (40 mg), or control blank silastic pellets were implanted subcutaneously in 2-month-old **(a) **BALB/c or **(b) **C57BL/6 female mice. After 2 months, the mice were killed and the mammary glands processed for histological evaluation (H&E) or for WM (as described in the Materials and methods section). A representative image observed in at least four different mice of each group is presented. The numbers of ducts (black arrow), lobules (white arrow), and lateral branching (dotted arrow) were **(c) **quantified in each H&E stained slide and expressed as the number of structures per 400× field. MPA treatment induced an increase in the number of ducts and in lateral branching only in BALB/c mice, whereas Pg induced an increase in the number of lobules mainly in BALB/c. In control groups, animals were euthanized at diestrus. ***P < 0.001, treated versus control; ^a^*P *< 0.05 between controls of both strains. Magnification: 100×, bar: 100 μm. H&E, hematoxylin and eosin; MPA, medroxyprogesterone acetate; Pg, progesterone; WM, whole mount.

To evaluate early morphologic responses to MPA or progesterone on the mammary gland, C57BL/6 and BALB/c mice were treated with MPA or progesterone, or implanted with blank silastic pellets for 4 or 8 weeks, after which the animals were killed and the mammary glands excised and processed for whole mount or hematoxylin and eosin studies. When untreated mammary glands from control virgin mice of both strains, without any treatment and in the same phase of the estrous cycle, were compared, BALB/c untreated mammary glands exhibited more ductal side branching than did C57BL/6 glands (Figure 2a,b, left panels), and only BALB/c mice exhibited a significant increase in MPA-induced ductal branching in mammary glands (Figure 2a,b, middle panels; *P *< 0.001). The effect of progesterone was different from that of MPA; lobular differentiation was observed in both strains, although the effect in BALB/c mice was more prominent (Figures 2a,b, right panels). Quantification of these morphologic differences is shown in Figure 2c. These experiments confirm that MPA and progesterone have different effects in C57BL/6 and BALB/c animals, with significantly lower hormone responsiveness in the C57BL/6 strain.

#### Hormone receptors

**Figure 3:**
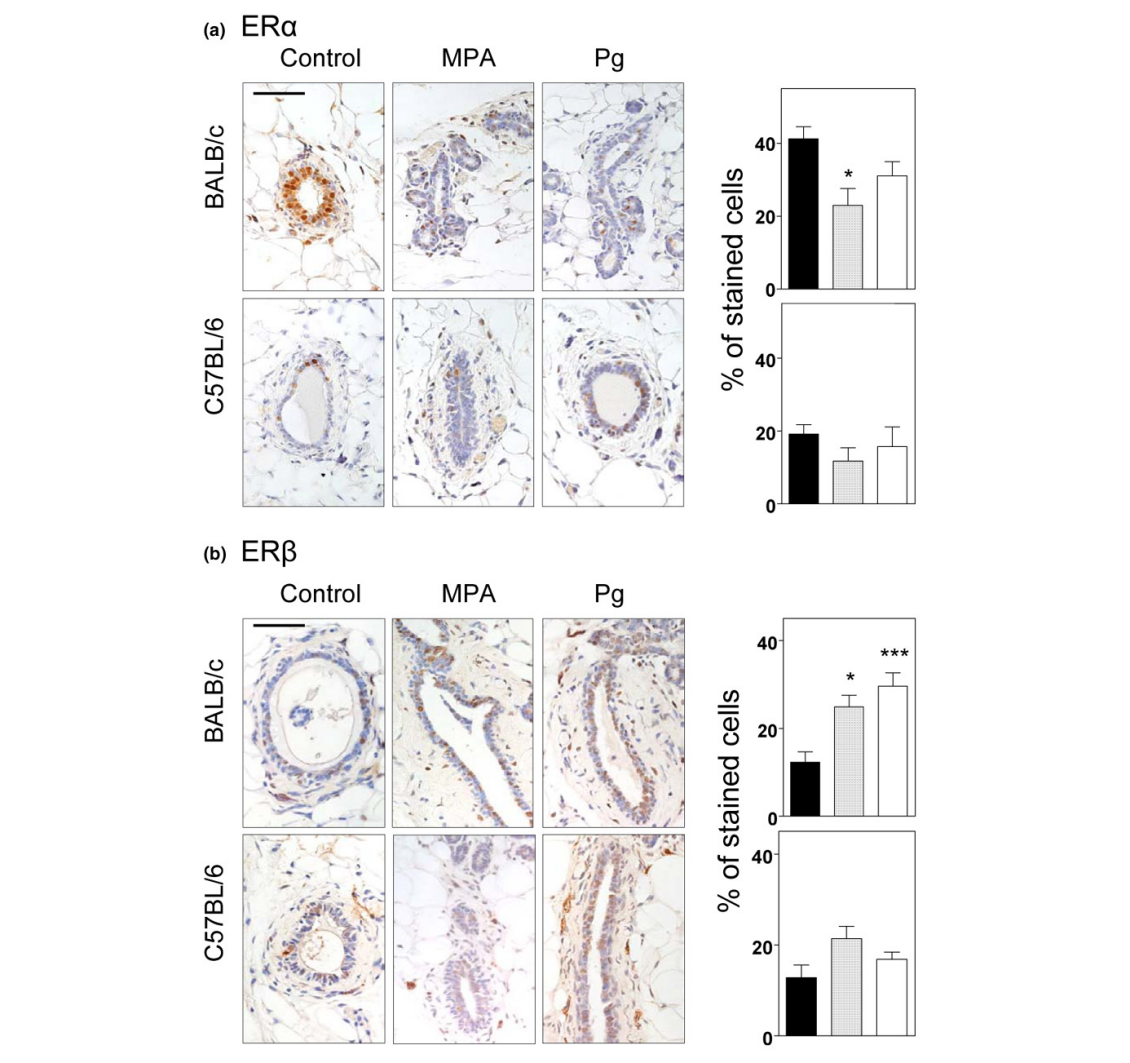
ER expression in mammary glands treated with progestins for 2 months. For both **(a) **ER-α and **(b) **ER-β, a representative image of four to 10 histological slides/group is shown. The number of stained cells was quantified and expressed as a percentage of the number of stained cells among epithelial cells in 400× fields of every slide (bar: 80 μm). A minimum of 1,500 epithelial cells/slide were counted. The right panels show the quantification in control (filled columns), MPA-treated (dotted columns), and Pg-treated (empty columns) mice (mean ± standard error). ER-α was significantly lower in C57BL/6 mice (*P *< 0.001), and there was a significant decrease with MPA treatment in BALB/c mice. Regarding ER-β, there was a significant increase in progestin-treated BALB/c mice. In control groups, animals were killed at diestrus; animals were treated for 2 months. *P < 0.05, ***P < 0.001 control versus treated mice. ER, estrogen receptor; MPA, medroxyprogesterone acetate; Pg, progesterone.

**Figure 4:**
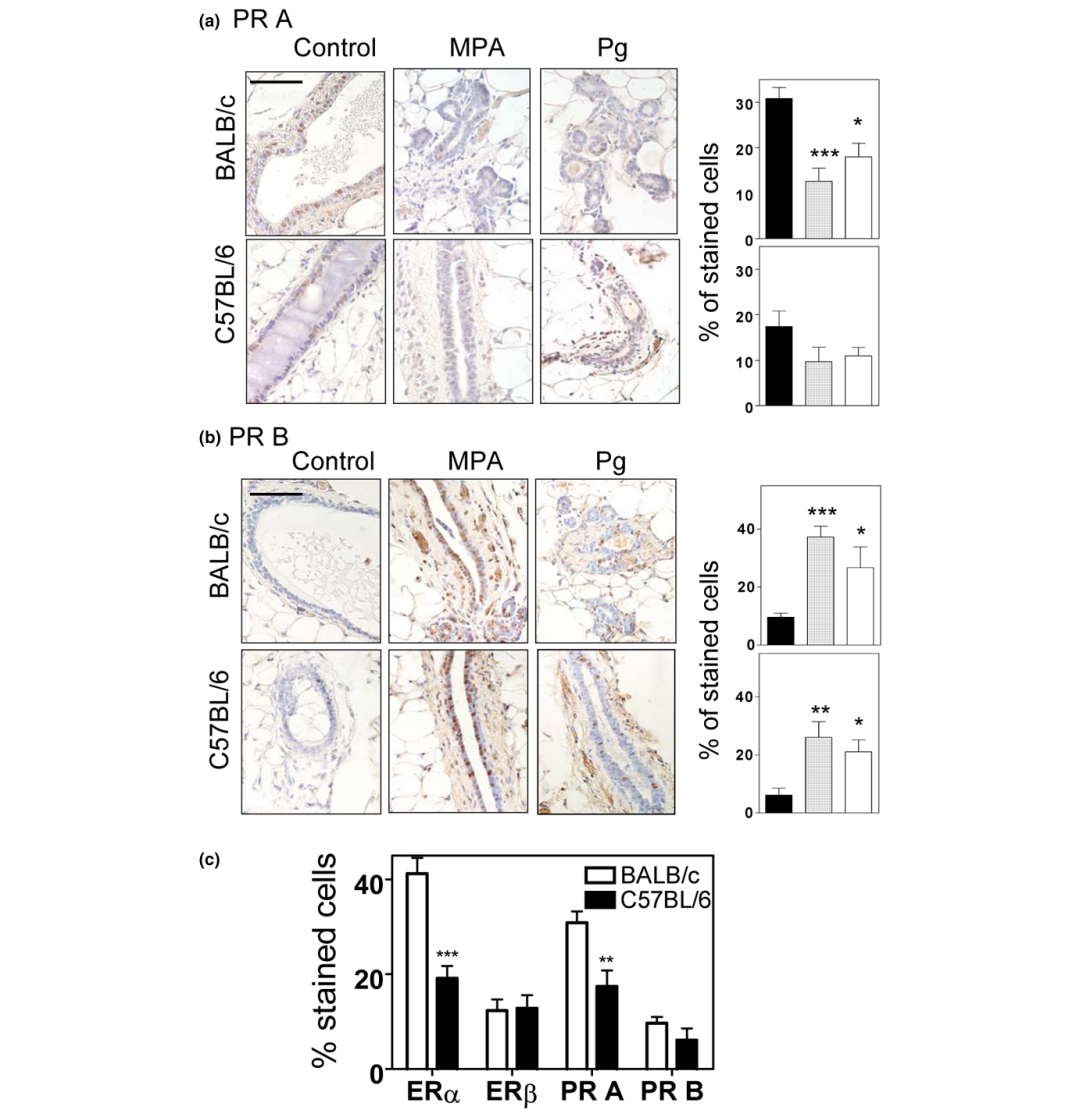
PR expression in mammary glands treated with progestins for 2 months: immunohistochemical studies. Control mice received blank silastic pellets. For both **(a) **PR-A and **(b) **PR-B, a representative image of four to six histologic slides per group is shown. The number of stained cells is expressed as the percentage of the number of stained cells per epithelial cells in 400× fields of every slide and a minimum of 1,500 epithelial cells/slide were counted (bar: 80 μm). The right panels show the quantification in control (filled columns), MPA-treated (dotted columns), and Pg-treated (empty columns) mice (mean ± standard error). PR-A expression decreased significantly in progestin-treated BALB/c mice, whereas PR-B expression increased in progestin-treated mice of both strains. *P < 0.05, **P < 0.01, ***P < 0.001 control versus treated mice. **(c) **Quantification (mean ± standard error) of the percentage of positive stained cells for ER-α, ER-β, PR-A, and PR-B in control BALB/c or C57BL/6 female mice shown in Figures 3 and 4a,b. BALB/c mice show higher levels of ER-α and PR-A than C57BL/6 female mice. In control groups, animals were killed at diestrus. ***P < 0.001, **P < 0.01. ER, estrogen receptor; MPA, medroxyprogesterone acetate; Pg, progesterone; PR, progesterone receptor.

All receptors were evaluated by immunohistochemistry using validated antibodies [35-38]. The number of positive cells was expressed as the percentage of stained nuclei per number of epithelial cells/400× fields. There were significant differences in the number of ER-α-positive cells between virgin mammary glands of both strains (41.2 ± 10% in BALB/c versus 19 ± 7.5% in C57BL/6 mice; *P *< 0.001; Figures 3a and 4c). MPA treatment resulted in a significant decrease in ER-α expression only in BALB/c mice (Figure 3a; *P *< 0.05). ER-β expression was low in control virgin mice of both strains (Figures 3b and 4c) that increased in MPA (*P *< 0.05) and in progesterone-treated BALB/c animals (*P *< 0.001; Figure 3b) but not in C57BL/6 mice.

**Figure 5:**
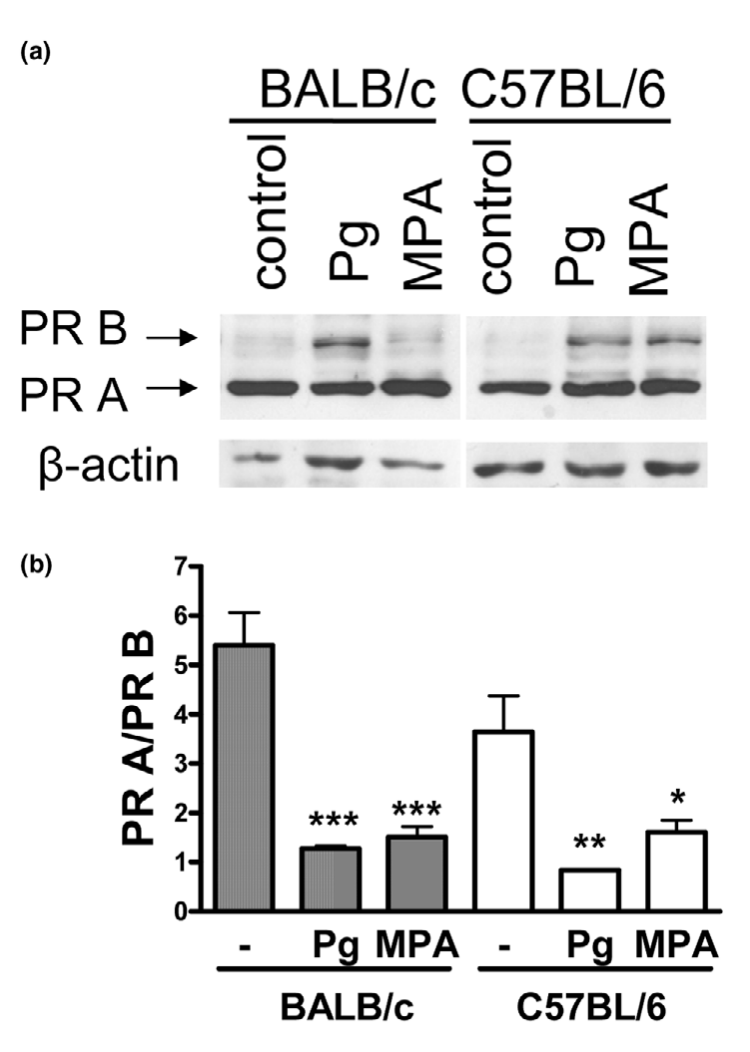
PR expression in mammary glands treated with progestins for 2 months: Western blot studies. Mammary glands from the same groups shown in Figure 4 were processed to obtain nuclear extracts (as explained in Materials and methods) and used in Western blots. **(a) **A representative experiment using C-19 PR Santa Cruz antibody is shown. Because different amounts of epithelial cells are present in the different extracts, the **(b) **ratio of PR-A/PR-B in three different experiments is shown. *P < 0.05, **P < 0.01, ***P < 0.001 treated versus control. PR, progesterone receptor.

The pattern of PR-A expression was very similar to that of ER-α; again, significantly higher levels were observed in untreated BALB/c mice (Figure 4a,c), which were more efficiently downregulated by MPA (*P *< 0.001) than by progesterone (*P *< 0.05; Figure 5a). Virgin untreated mice of both strains exhibit almost no PR-B expression, as previously reported [35] (Figures 4b,c). Interestingly, PR-B was highly expressed in MPA and progesterone treated BALB/c (*P *< 0.001 and *P *< 0.05, respectively) and C57BL/6 mice (*P *< 0.01; Figure 4b), and followed a similar pattern of expression as ER-β.

Nuclear extracts from progestin-treated or virgin control mice were used for Western blots (Figure 5a). Extracts were normalized by protein content. Because mammary glands from virgin mice have less epithelial cells than progestin-treated mice, and on the other hand both stroma and epithelium may express steroid receptors, which may be differentially regulated, we only quantified the ratio between both isoforms in the same blot (Figure 5b). The PR-A/PR-B ratio was significantly higher in virgin as compared with progestin treated mice of both strains, supporting the immunohistochemical data. In addition, virgin BALB/c mammary glands expressed higher levels of PR-A and ER-α (data not shown) than did C57BL/6 glands (*P *< 0.01). Significant differences between MPA and progesterone treatment were not detected in Western blots.

#### Progestin effect on salivary gland morphology

**Figure 6:**
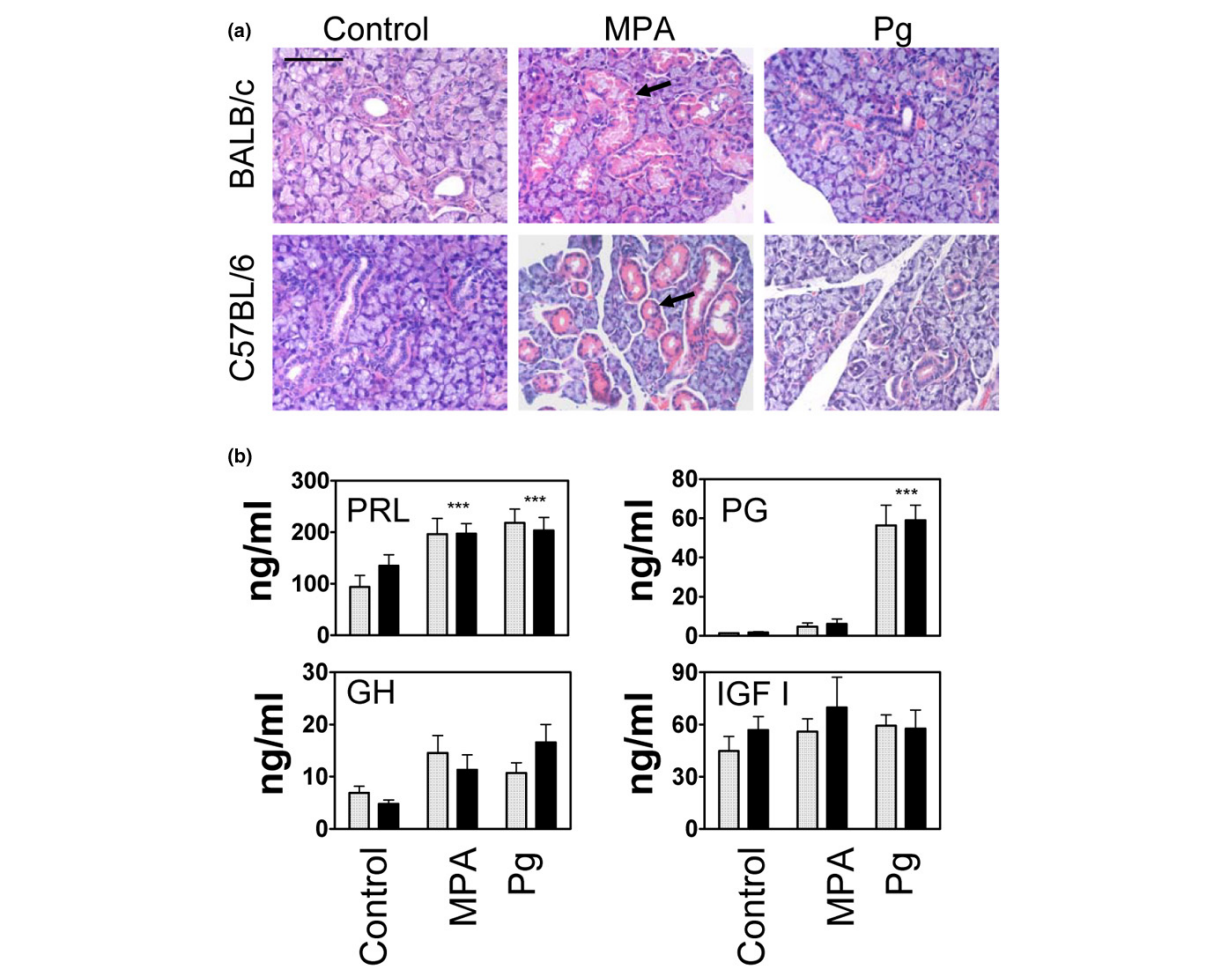
Effects of progestins in salivary gland morphology and in serum pituitary hormone levels. **(a) **Salivary glands from control, MPA, or Pg-treated BALB/c or C57BL/6 mice. Pellets were implanted subcutaneously and the animals killed after 2 months. MPA increased the development of convoluted granular ducts (arrow). No differences were observed between both strains of mice (400×, bar: 120 μm). **(b) **Serum PRL, Pg, GH, and IGF-I levels measured by radioimmunoassay in control BALB/c or C57BL/6 female mice and after 24 hours of MPA or Pg treatment. No differences were detected in C57BL/6 mice as compared with BALB/c mice. Furthermore, Pg and MPA treatment induced an increase in PRL levels in both strains (*P *< 0.001, two-way ANOVA). The increase in Pg levels after progesterone administration was also similar in both strains (*P *< 0.001, two-way ANOVA). Gray bars: BALB/c; black bars: C57BL/6. ANOVA, analysis of variance; GH, growth hormone; IGF, insulin-like growth factor; MPA, medroxyprogesterone acetate; Pg, progesterone; PR, progesterone receptor; PRL, prolactin.

MPA, because of its androgenic action [39], has a specific effect on salivary gland morphology in that it induces the development of convoluted granular ducts [24], which was observed in both strains (Figure 6a). Progesterone had no notable effects on salivary glands.

#### Serum prolactin, growth hormone, progesteron, and insulin-like growth factor-I levels

ER knockout mice develop a hypoplastic mammary gland phenotype as a consequence of a pituitary gland dysfunction associated with a decrease in prolactin levels [40]. To explore the possibility of pituitary dysfunction as an explanation for the strain differences in hormone responsiveness, we evaluated serum levels of prolactin in control and progestin-treated mice, but we failed to observe any difference between C57BL/6 and BALB/c mice. In addition, prolactin levels were increased in progestin-treated animals of both strains (*P *< 0.001, two-way ANOVA; Figure 6b). Similarly, no differences in progesterone, growth hormone, or IGF-I levels between strains were observed. Furthermore, serum levels of progesterone reached after the implantation of 20 mg progesterone pellets was also similar in both strains (Figure 6b).

### Transplantation experiments

**Figure 7:**
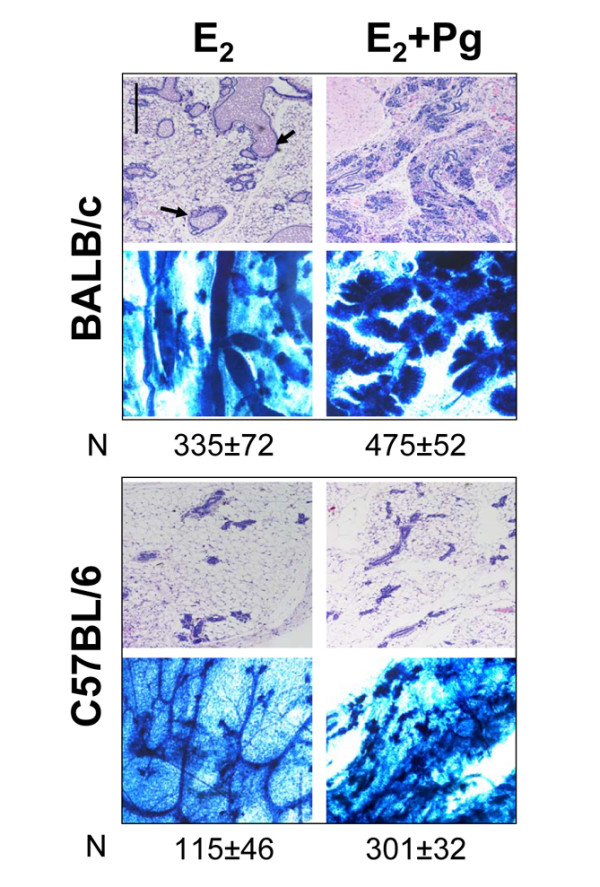
Mammary glands from BALB/c or C57BL/6 mice treated with E_2 _or E_2 _plus Pg. Mice were implanted subcutaneously with silastic pellets containing E_2 _(20 μg) or Pg (20 mg) + E_2 _(20 μg). Representative H&E stained sections and WM are shown. The arrow points to ductal dilatations, typical of E_2 _action in BALB/c mice. All WM pictures were taken at the same magnification. Magnification: 100×, bar: 1.1 μm.*P *< 0.05, BALB/c versus C57BL/6. E_2_, 17β-estradiol; H&E, hematoxylin and eosin; N, number of structures/slide; Pg, progesterone; WM, whole mount.

**Figure 8:**
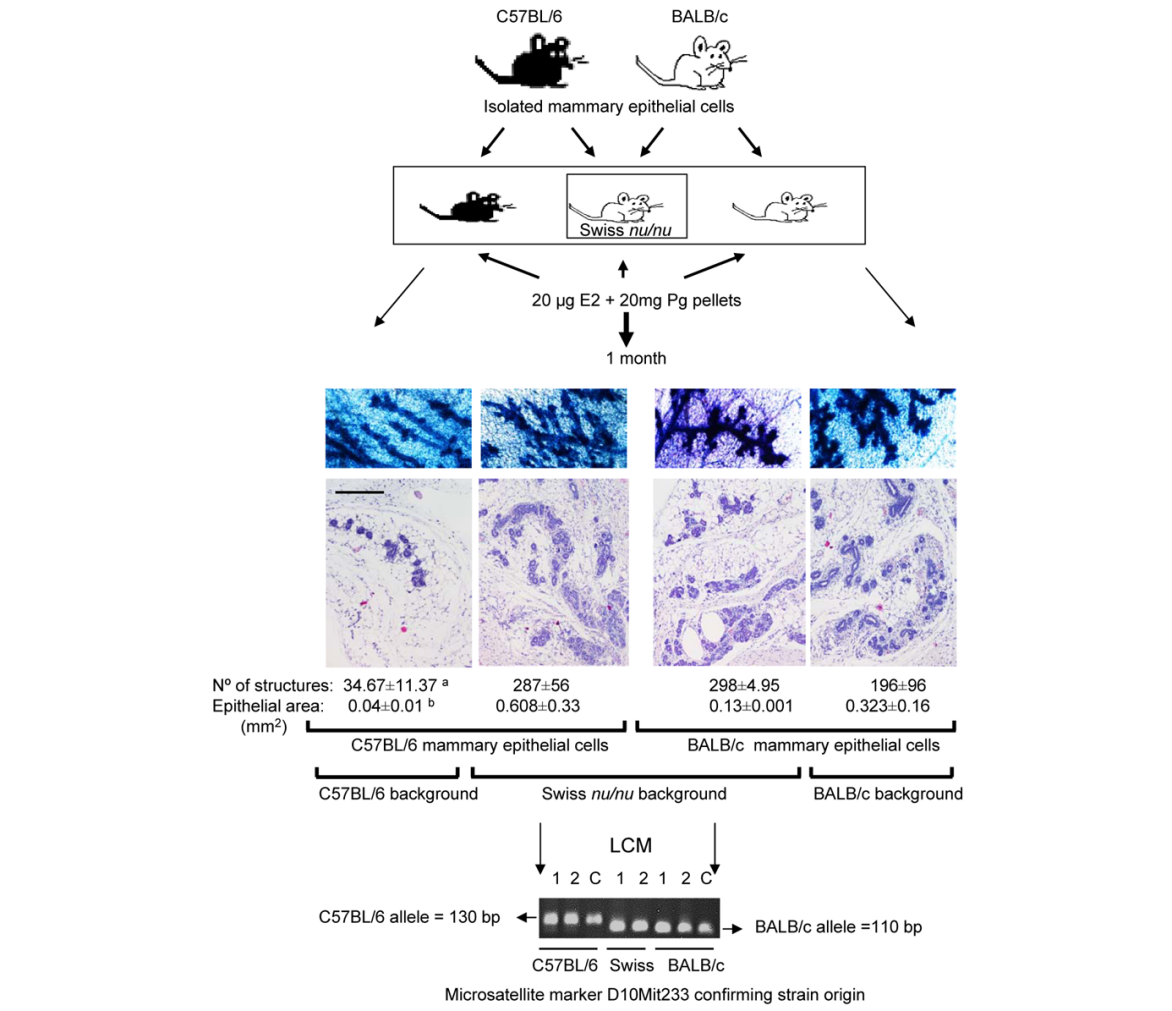
Morphology of reconstituted mammary glands of both strains into a *nude *background. Mammary cells were isolated from 2-month-old BALB/c or C57BL/6 mice and 5 μl of cell suspensions were transplanted into the right or left cleared mammary fat pads, respectively, of 21-day-old Swiss *nu/nu *or syngeneic mice. At surgery pellets containing E_2 _plus Pg or plus MPA were also implanted subcutneously into the backs of the animals. One month later, animals were killed and mammary glands excised and fixed. Microscopic examination revealed no changes between C57BL/6 or BALB/c repopulated Swiss *nu*/*nu *glands, whereas C57BL/6 cells transplanted in syngeneic glands were under-developed (100×, bar: 700 μm). A representative image of the WM or the H&E stained slides of the reconstituted mammary glands in E_2_/Pg-treated mice is shown. Microsatellite studies confirmed the strain origin of the epithelial cells in the Swiss background. bp, base pairs; E_2_, 17β-estradiol; H&E, hematoxylin and eosin; LCM, laser capture microdissection; MPA, medroxyprogesterone acetate; Pg, progesterone; WM, whole mount.

To further explore whether the responsiveness of this strain was related to differences in the epithelial mammary cells or in the stromal environment, we transplanted the epithelial mammary cells into *nu/nu *mice of a different strain background. Because nude mice lack functional ovaries and consequently have very low E_2 _levels, this hormone had to be supplemented (20 μg) to the MPA or progesterone standard pellets. Therefore, we first evaluated whether the differences between strains were also observed when E_2 _or combined E_2 _and progesterone pellets were used. The differences were significant, even with E_2 _alone (Figure 7). E_2 _induced duct dilation whereas the combined treatment induced a high proliferative response, with increased branching and lobule-alveolar differentiation. Surprisingly, however, C57BL/6 cells were able to repopulate the cleared fat pads of MPA/E_2_-treated or progesterone/E_2_-treated Swiss *nu*/*nu *mice as efficiently as BALB/c cells (Figure 8). In contrast, BALB/c cells fared better in repopulating BALB/c cleared fat pads than did C57BL/6 cells in C57BL/6 cleared fat pads.

To confirm that the transplanted cells were those giving rise to the mammary glands and not some pre-existing epithelial cells from the recipient Swiss *nu*/*nu *female, ducts and lobules were excised by laser capture microdissection and genotyped by PCR using four informative microsatellite markers (D7Mit69, D7Mit82, D10Mit233, and D13Mit7). These markers were selected to be polymorphic between C57BL/6 and the recipient *nude *females. The PCR analysis clearly showed that the transplanted epithelial cells excised by laser capture microdissection were C57BL/6 in origin (Figure 8). These results indicate that strain-specific host factors probably modulate mammary epithelial hormone responsiveness.

#### ER and PR expression in recombined mammary glands in *nu/nu *mice

**Figure 9:**
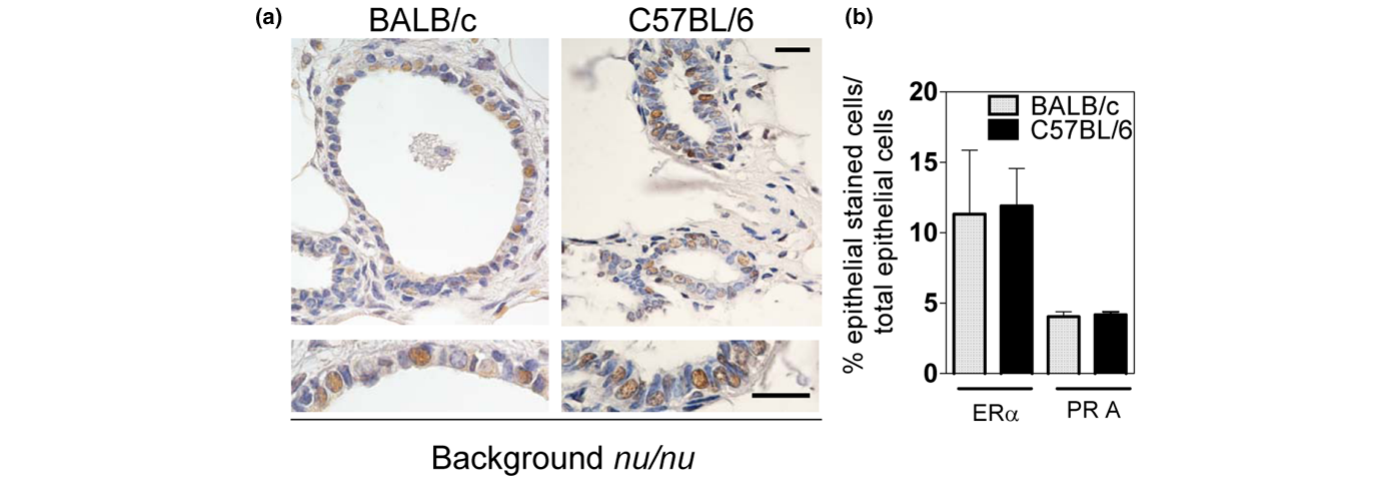
ER-α expression in reconstituted mammary glands. Epithelial mammary cells of BALB/c or C57BL/6 mice were purified and implanted into cleared fat pads of untreated *nu*/*nu *mice (as explained in Materials and methods). After 1 month, mammary glands were excised and fixed for histological evaluation. **(a) **ER-α was evaluated using MC 20 Santa Cruz antibody and PR-A (not shown) using C-20 Santa Cruz antibody (bar: 45 μm). **(b) **The number of stained cells is expressed as the percentage of stained cells among epithelial cells in 400× fields of every slide. ER, estrogen receptor; PR, progesterone receptor.

We were now interested in determining whether the differences in ER-α and PR-A expression observed between both strains would even out when epithelial mammary cells from both strains were inoculated in the same host. Because the differences were evident only in untreated animals, we performed the same recombination experiments in untreated mice. Figure 9 shows ducts that originated from C57BL/6 or BALB/c cells transplanted in the same *nu*/*nu *mouse; no significant differences in ER-α or PR-A expression were observed. These results indicate that host factors are responsible for the strain differences observed in mammary gland hormone receptor expression and that the level of these receptors is related to the degree of hormone responsiveness.

## Discussion

The C57BL/6 strain is reputed to be relatively resistant to some chemical carcinogenesis protocols [41-43] and relatively susceptible to spontaneous and chemically induced melanomas [44], radiation-induced leukemia [45], and chemically induced urinary bladder carcinomas [46]. Regarding mammary tumorigenesis, the C57BL/6 strain is considered to be resistant to MMTV [7], urethane [47], and γ radiation [20,21].

In this study we demonstrated that C57BL/6 female mice were resistant to MPA-induced carcinogenesis, and that this resistance was associated with poor mammary gland responsiveness to the exogenous administration of progestins and/or estrogens. We also observed significantly different levels of ER-α and PR-A in the sensitive and resistant strains, that may be associated with the poor responsiveness described. Surprisingly, C57BL/6 mammary epithelial cells may be as responsive as BALB/c cells and exhibit similar levels of hormone receptors when transplanted into cleared fat pads of a different strain, indicating that host factors rather than the epithelial cells by themselves are responsible for their hormone resistance.

A hypoplastic phenotype of C57BL/6 mammary glands was reported by Nandi and Bern in 1960 [48]. In most of their experiments, hormonal treatment was provided by the kidney subcapsular implantation of the pituitary gland, or by the administration of estrogens or combinations of estrogens plus progesterone; the effects of progestins alone, however, have been evaluated in very few studies [23,49]. Our findings demonstrate a differential effect of progestins in BALB/c and C57BL/6 female mice and confirm the observations by Nandi and Bern regarding differences in mammary glands of virgin mice. MPA induced ductal branching whereas progesterone induced lobular differentiation in BALB/c and not in C57BL/6 mice. These observations correlate with the fact that MPA induced mainly ductal carcinomas whereas progesterone generated mainly lobular carcinomas in BALB/c mice [23]. These tumors are different not only in histology but also in ER, PR, and epidermal growth factor (EGF) receptor expression [24].

Recent studies indicate that progesterone and MPA trigger different transduction signals in the human breast cancer cell line T47D [50] and in human umbilical vein endothelial cells [51]. Different *in vivo *effects were also reported in previous studies, in which it was observed that MPA induced the synthesis of EGF in salivary glands as an androgen receptor ligand [39,52]. This effect was not achieved by progesterone. As we have demonstrated in previous studies, salivary gland EGF does participate in MPA-induced mammary gland carcinogenesis [24,53]. Because C57BL/6 mammary glands responded poorly to both MPA and progesterone, it seemed unlikely that the differences in the two strains were related to differences in salivary gland responsiveness. This was supported by the fact that no histologic differences with respect to MPA-induced development of convoluted granular ducts of the salivary glands, the source of EGF, were found across strains. MPA has been used as an anabolic agent in oncology [54] and, as we have shown in previous studies, it exerts an anabolic effect in BALB/c female mice [33]. In the present study we demonstrated a similar effect in C57BL/6 mice, showing that this is a strain-independent mechanism.

We have also explored the possibility of impaired pituitary function, which plays a very important role in determining the hypoplastic mammary gland phenotype in ER knockout mice [40], and is associated with low prolactin levels. However, in our study, prolactin levels were similar in C57BL/6 and BALB/c mice and in progestin-treated animals of both strains. The same was true for progesterone, growth hormone, and IGF-I serum levels.

Taken together, all of the described results appeared to point to strain differences intrinsic to the mammary gland. To explore this possibility, we sought to determine the presence and functionality of mammary steroid receptors in both strains. To evaluate PR A, we used C-19 and C-20 antibodies (Santa Cruz) that recognize this isoform in human tissues [55] and mouse tissues [35,38]. We observed significantly lower levels of ER-α and PR-A in virgin C57BL/6 adult female mice, as compared with BALB/c. The only PR isoform that we found to be highly expressed in BALB/c virgin mice was PR-A, as was previously described by Auperlee and coworkers [35]. PR-A and ER-α were significantly downregulated in both BALB/c MPA-treated and progesterone-treated mice, and it is interesting to note that ER-α expression usually paralleled that of PR-A. ER-β and PR-B exhibited similar kinetics; their expression was low in control mice and it increased in progestin-treated BALB/c mice. Aupperlee and coworkers [35] reported similar results in their studies of PR-B expression in pregnant BALB/c mice.

These results, when extrapolated to humans, suggest that individual levels of ER-α and PR-A in the mammary glands could predict hormone responsiveness and cancer risk. It has been reported that low levels of ER-α expression are observed in women of low risk groups [56,57]. As proof of principle, Frech and coworkers [58] developed transgenic mice that overexpress ER-α in a C57BL/6 background, in which the number of ER-α positive cells in transfected glands was similar to that in the virgin BALB/c mice we report on here. Curiously, ductal hyperplasias were reported in the transgenic mice even in the absence of estrogens. As a consequence of ER-α overexpression, increased PR levels were also observed.

The expression of ER and PR is regulated by endocrine factors and by extracellular matrix components such as collagen IV or laminin [59]. To explore a role for the stroma in the strain difference in hormone responsiveness, we used transplanted epithelial cells into the cleared fat pad of Swiss nude mice. The fact that both C57BL/6 and BALB/c epithelial cells responded very similarly in a different strain background suggests that the mammary gland fat pad from the host may be regulating the epithelial hormone responsiveness. At this moment we cannot, however, rule out the participation in the process of other host factors related to the microenvironment, which may be also be playing an active role. Experiments are currently underway in our laboratory to test this alternative hypothesis. Similar data were reported by Naylor and Ormandy [60]. Those investigators recombined epithelial cells from 129 strain, which is similar to BALB/c, with C57BL/6 stroma, and observed a poorly side-branched C57BL/6 pattern. A major challenge will be to identify the stromal factors responsible for maintaining high ER and PR-A levels in BALB/c mice.

Different studies have been designed to explain BALB/c susceptibility to mammary carcinogenesis. The frequency of mammary tumors differs among strains of *Trp53*^+/- ^mice, with mammary tumors occurring only on a BALB/c genetic background and showing a high frequency of loss of heterozygosity [61]. Interestingly, progesterone is necessary and favors aneuploidy in this model [62]. In the irradiation model, the increased genomic instability of BALB/c mice has been ascribed to two polymorphisms in the coding region of *Prkdc *[63]; this is the gene that encodes the DNA-dependent protein kinase catalytic subunit, which is known to be involved in DNA double-stranded break repair. Because it has been shown that the stroma may be a target in radiation-induced [64] and in chemical-induced carcinogenesis [65], other players important in parenchymal-stromal interactions related with hormone receptor expression may be involved in all models.

## Conclusion

In this study we demonstrated impaired hormone responsiveness in C57BL/6 mammary epithelial cells that is reflected in responses to both physiologic and neoplastic stimuli, making the strain resistant to hormonal carcinogenesis. This was associated with low levels of ER-α and PR-A. Further exploring the phenomenon, we demonstrated that this is not due to intrinsic impairments in the epithelial cells and that host factors, probably related to the mammary microenvironment, may account for the defective hormonal behavior. The comparative study of C57BL/6 and BALB/c mice may help us to understand the genetic basis of resistance and susceptibility to hormone-induced mammary carcinogenesis.

## Abbreviations

ANOVA = analysis of variance; E_2 _= 17β-estradiol; EGF = epidermal grwoth factor; ER = estrogen receptor; IGF = insulin-like growth factor; MPA = medroxyprogesterone acetate; MMTV = murine mammary tumor virus; NIDDK = National Institute of Diabetes and Digestive and Kidney Diseases; PBST = phosphate-buffered saline-Tween; PCR = polymerase chain reaction; PR = progesterone receptor;

## Competing interests

The authors declare that they have no competing interests.

## Authors' contributions

GMG and SV carried out all the immunohistochemical and morphologic studies. JPC and MCB conducted all of the transplantation experiments in nude mice. JB conducted all of the *in vivo *experiments to test the carcinogenic effect of MPA and the whole-mount studies in the long-term experiments. JV and DBV carried out the radioimmunoassay. FB conducted the microsatellite analysis. VP and MCB conducted the laser capture microdissectionanalysis in the SG Laboratory. CL and AM designed, coordinated and drafted the manuscript. All authors read and approved the final manuscript.
